# Inclisiran-Induced Fixed Drug Eruption in a Patient With Refractory Hypercholesterolemia

**DOI:** 10.7759/cureus.98687

**Published:** 2025-12-08

**Authors:** Jawad A Khan, Shahab Shahab

**Affiliations:** 1 Geriatric Medicine, Midland Metropolitan University Hospital, Birmingham, GBR; 2 Cardiology, Sandwell and West Birmingham Hospitals NHS Trust, Birmingham, GBR

**Keywords:** bempedoic acid, cutaneous adverse reaction, fixed drug eruption, hypercholesterolemia, inclisiran, lipid-lowering therapy, pcsk9 inhibition, statin intolerance

## Abstract

We report the case of a 72-year-old man with a history of atrial fibrillation, hypertension, and refractory hypercholesterolemia who developed a fixed drug eruption following treatment with inclisiran, a small interfering RNA (siRNA) targeting proprotein convertase subtilisin/kexin type 9 (PCSK9). The patient had documented statin intolerance and inadequate response to ezetimibe and bempedoic acid, prompting initiation of inclisiran. Following the second injection, he developed a persistent pruritic erythematous rash affecting the lower abdomen and legs. Dermatologic assessment, including skin biopsy, confirmed a drug eruption. Symptoms improved with topical therapy and antihistamines. Due to the ongoing need for lipid control, bempedoic acid was reintroduced without recurrence of the rash. This case highlights a rare cutaneous adverse reaction to inclisiran and underscores the importance of monitoring for dermatologic complications associated with novel lipid-lowering therapies.

## Introduction

Hypercholesterolemia, or high levels of cholesterol in the blood, is a major contributor to atherosclerotic cardiovascular disease. Statins are the first-line therapy for lowering cholesterol, but some patients experience statin intolerance, which refers to adverse effects that prevent continued use or do not achieve adequate low-density lipoprotein (LDL) cholesterol reduction despite therapy. Inclisiran is a novel small-interfering RNA (siRNA) therapy that targets proprotein convertase subtilisin/kexin type 9 (PCSK9), a protein that regulates LDL cholesterol levels. By silencing the PCSK9 gene, inclisiran reduces LDL cholesterol in the bloodstream. Clinical trials report that the most frequent adverse effects are mild injection-site reactions, with few descriptions of generalized or persistent cutaneous reactions. Fixed drug eruptions (FDEs) are a type of skin reaction that recurs at the same site upon exposure to a medication and can present as red, itchy, or inflamed patches.

We present a rare case of an FDE associated with inclisiran in a patient with refractory hypercholesterolemia, highlighting the importance of recognizing atypical dermatologic reactions in patients receiving novel lipid-lowering therapies.

## Case presentation

A 72-year-old male with a history of hypertension, atrial fibrillation, and refractory hypercholesterolemia was evaluated in May 2021 for dizziness. Holter monitoring revealed nocturnal bradycardia and sinus pauses. His lipid testing showed persistent hypercholesterolemia, with total cholesterol ranging between 5.2 and 5.9 mmol/L. The patient had documented statin intolerance and reported inadequate responses to both ezetimibe and bempedoic acid. At the time of presentation, he had been taking losartan and bisoprolol for hypertension, and apixaban for atrial fibrillation, for over two years without any dermatologic reactions. None of these medications was suspected to contribute to the FDE. Table 1 summarizes the patient's lipid profile over time.

**Table 1:** Lipid profile summary. Laboratory reference ranges are provided for each lipid parameter, noting that these values may vary slightly between laboratories. All patient values are reported in mmol/L.

Date	Total cholesterol (mmol/L)	HDL (mmol/L)	LDL (mmol/L)	Triglycerides (mmol/L)
Reference range	3.6-5.2	1.0-1.5	<3.0	<1.7
03/2020	5.2	1.6	-	1.6
06/2021	6.0	1.7	-	1.1
04/2022	5.2	1.6	-	1.0
06/2022	4.7	1.3	0.8	-
10/2022	5.8	1.6	-	1.0
05/2023	4.2	1.6	-	1.1
12/2023	3.7	1.7	1.5	1.0

**Figure 1:**
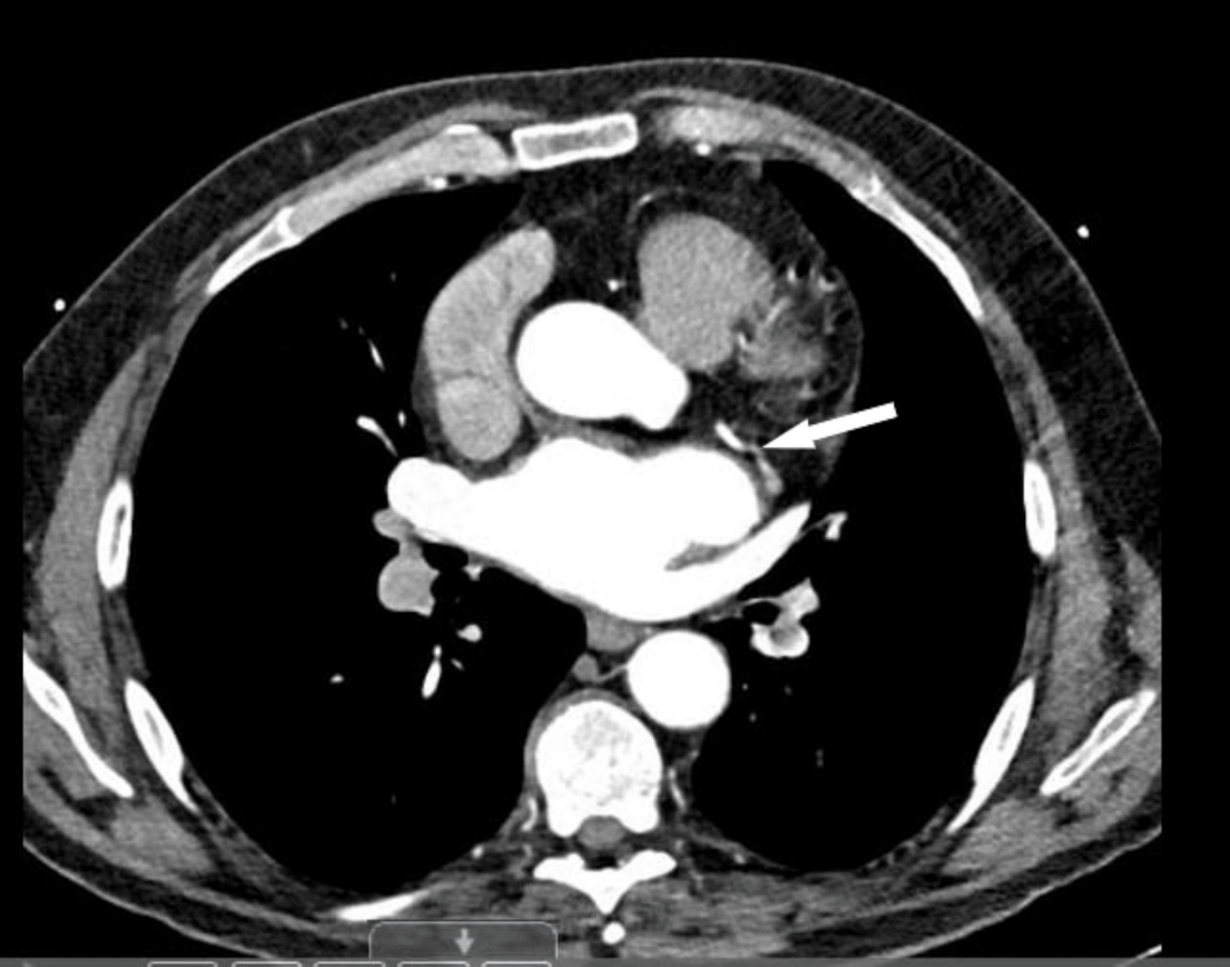
CT coronary angiogram showing moderate atherosclerosis in the left circumflex artery (LCx). The white arrow indicates the lesion.

In June 2021, ezetimibe (10 mg daily) was initiated, but his cholesterol remained elevated at 6.0 mmol/L. Bempedoic acid (180 mg daily) was added, but LDL levels remained persistently high. Given the patient’s refractory hypercholesterolemia and multiple cardiovascular risk factors, a CT coronary angiogram was performed to assess the extent of coronary atherosclerosis. This revealed moderate atherosclerosis in the left circumflex artery (LCx), as shown in Figure 1. There was no significant stenosis in the right coronary artery (RCA) or left anterior descending artery (LAD).

**Figure 2:**
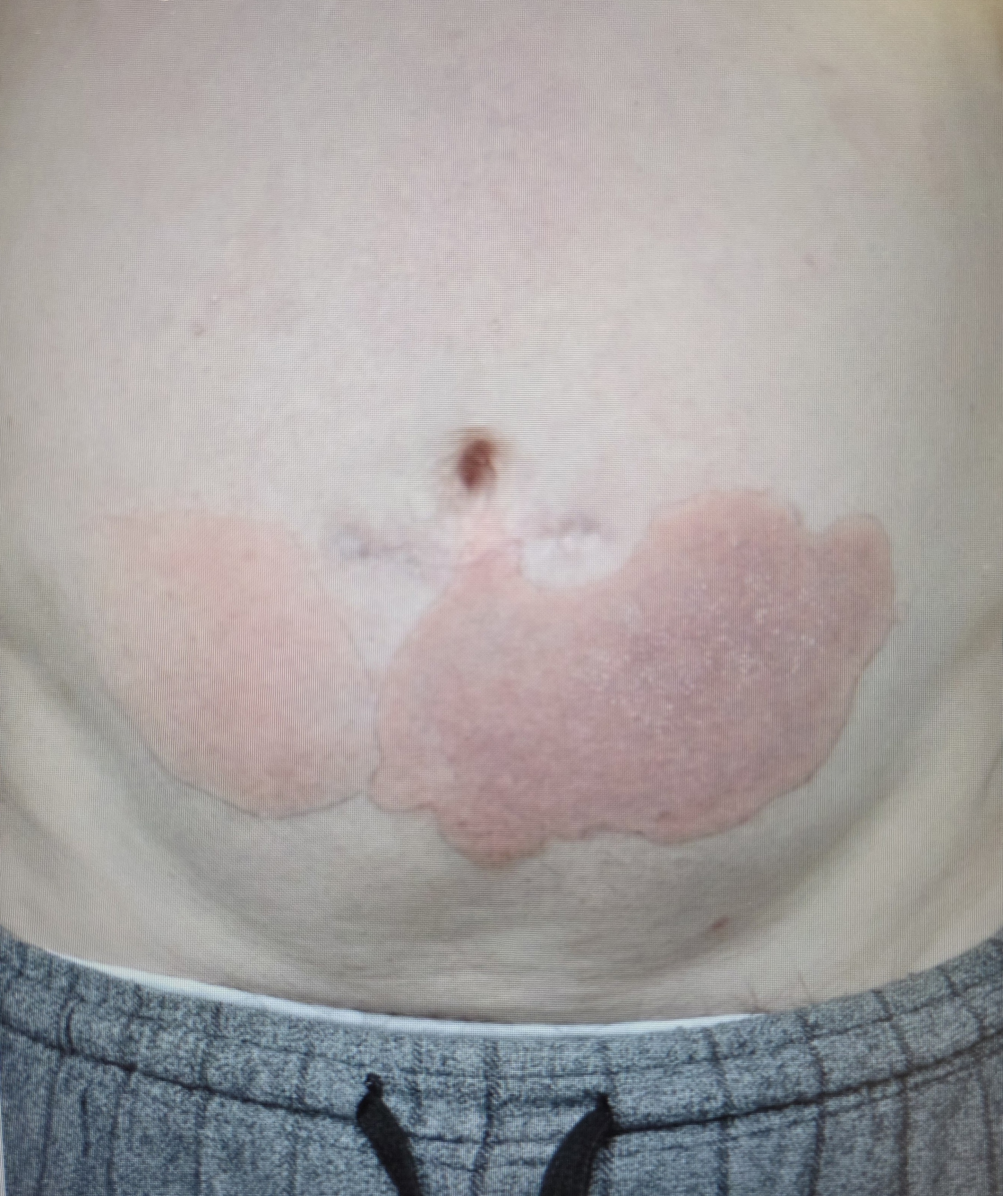
Well-demarcated, irregular erythematous patches over the lower abdomen, showing areas of confluent redness with mild scaling. The lesions are non-indurated and exhibit a fixed distribution, consistent with a fixed drug eruption. No ulceration or vesiculation is visible.

Despite combination therapy, LDL cholesterol remained elevated at 3.4 mmol/L, prompting the initiation of inclisiran in January 2023. After the second injection in April 2023, the patient developed a pruritic erythematous rash on the lower abdomen and legs within several days. The rash became progressively uncomfortable and painful, as seen in Figure 2.

Topical Cetraben cream and loratadine provided partial relief, but Betnovate RD worsened the irritation. Dermatologic evaluation revealed well-demarcated, irregular erythematous patches over the lower abdomen with areas of confluent redness and mild scaling, consistent with an FDE. Skin biopsy performed on June 26, 2023, confirmed superficial perivascular inflammation and folliculitis. These findings aligned with the histopathological patterns typically observed in FDEs, which often include epidermal necrosis, basal cell vacuolar change, and perivascular lymphocytic infiltrates. In this patient, the timing of rash onset after inclisiran administration, its fixed distribution, and supportive biopsy findings strongly supported the diagnosis of FDE over other dermatologic conditions. There were no signs of ulceration or vesiculation, and the eruption improved with topical corticosteroids, emollients, and antihistamines.

Due to the ongoing need for lipid control (with LDL levels reducing to 1.5 mmol/L in December 2023), bempedoic acid was safely reintroduced without recurrence of the rash. The patient continues follow-up with cardiology and reports a lipoprotein(a) level of 800 nmol/L.

Written informed consent was obtained from the patient for the publication of this case report and accompanying images.

## Discussion

Inclisiran is widely considered safe and effective, with adverse events typically limited to mild injection-site reactions [1,2]. Cutaneous events occurring away from the administration site are rare. FDEs are characterized by round or oval erythematous patches that recur at the same site after re-exposure to the causative drug [3].

In this case, the timing of symptom onset after the second inclisiran injection, along with supportive histopathological findings, strongly suggests an FDE attributable to inclisiran. Management involved topical corticosteroids, emollients, and antihistamines, which resulted in gradual resolution. Bempedoic acid was safely reintroduced, supporting inclisiran as the likely culprit.

As newer lipid-lowering agents such as inclisiran become more widely used, clinicians should be aware of rare but clinically relevant adverse dermatologic reactions, particularly in patients with limited therapeutic alternatives.

## Conclusions

This case reports a rare cutaneous adverse reaction to inclisiran, presenting as an FDE. Although based on a single patient, it underscores the need for clinicians to remain vigilant for atypical dermatologic reactions as the use of inclisiran expands in managing refractory hypercholesterolemia. Early recognition, timely dermatology input, and careful reassessment of lipid-lowering options are important to ensure patient safety and continuity of therapy. Reporting such events may help improve understanding of the safety profile of emerging lipid-lowering agents.

## References

[1] Ray KK, Wright RS, Kallend D (2017 ). Inclisiran in patients at high cardiovascular risk with elevated LDL cholesterol. N Engl J Med.

[2] Raal FJ, Kallend D, Ray KK (2020). Inclisiran for the treatment of heterozygous familial hypercholesterolemia. N Engl J Med.

[3] Korkij W, Soltani K (1984). Fixed drug eruption. A brief review. Arch Dermatol.

